# Single- or dual-antiplatelet therapy after transcatheter aortic valve replacement: A protocol of systematic review and meta-analysis

**DOI:** 10.1097/MD.0000000000024550

**Published:** 2021-02-12

**Authors:** Hongyan Li, Yafeng Wang, Longyu Li, Bao Dan

**Affiliations:** Qinghai People's Hospital, Qinghai, China.

**Keywords:** dual-antiplatelet therapy (DAPT), meta-analyses, randomized controlled trials, single-antiplatelet therapy (SAPT), systematic review, transcatheter aortic valve replacement (TAVR)

## Abstract

**Background:**

The evidence related to bleeding and thromboembolic events after transcatheter aortic valve replacement (TAVR) compared single antiplatelet therapy (SAPT) with dual antiplatelet therapy (DAPT) treatment are inconsistent. Moreover, there are some limitations such as small sample size and the risk of bias in existing studies. We will conduct a comprehensive systematic review and meta-analysis to explore the safety and efficacy of SAPT or DAPT after TAVR.

**Methods:**

A comprehensive literature search of PubMed, EMBASE, The Cochrane Library, Cochrane Central Register of Controlled Trials will be searched to retrieve studies involving SAPT versus DAPT after TAVR. Two investigators will independently select studies, extract data, and assess the quality of the included study. Any disagreement will be resolved by the third investigator. The study will use a random-effects model to pool the results of all studies and use the relative risk and 95% confidence intervals to summarize individual trial outcomes and estimate pooled effect. The study will use the Grading of Recommendations Assessment, Development, and Evaluation to assess the certainty of evidence.

**Results:**

This study will provide high-quality evidence for treatment of TAVR in terms of effectiveness and safety.

**Conclusion:**

This systematic review aims to provide evidence for treatment of TAVR in different antiplatelet therapies.

**Registration:**

The systematic review and meta-analysis is registered in the OSF REGISTRIES (10.17605/OSF.IO/Q42TE) international prospective register.

## 1 Introduction

Since the first transcatheter aortic valve replacement (TAVR) procedure in 2002, more than 300,000 procedures have been performed worldwide until mid-2016,^[1]^ and TAVR has been established as the first-choice treatment for many patients with severe aortic stenosis, especially in those at prohibitive or high surgical risk but also in an increasing number of patients at intermediate risk.^[2–6]^ Patients undergoing TAVR have a high incidence of hemorrhagic and thromboembolic events, not only during the perioperative period but also in the mid and long terms after the procedure.^[7–9]^

Practice guidelines recommend clopidogrel in addition to aspirin for the first 3 to 6 months after TAVR in patients who do not have an indication for oral anticoagulation.^[2,10]^ In trials that assessed the incidence of ischemic events after coronary-artery stenting, this form of dual-antiplatelet therapy was shown to reduce the risk of thromboembolic complications.^[11]^ However, a randomized controlled trial involving 665 patients after TAVR showed that the risk ratio of bleeding event was 0.57 in aspirin alone versus aspirin plus clopidogrel, and thromboembolic events at 1 year were significantly less frequent with aspirin.^[12]^ A systematic review suggested that compared with aspirin only, aspirin with clopidogrel was not shown to be superior in reducing thrombotic events, but presented a 3 times risk of major/life threatening bleeding.^[13,14]^

The evidence related to bleeding and thromboembolic events after TAVR compared single-antiplatelet therapy (SAPT) with dual-antiplatelet therapy (DAPT) treatment are inconsistent. Moreover, there are some limitations such as small sample size and the risk of bias in existing studies. Therefore, we designed a comprehensive systematic review and meta-analysis to explore the safety and efficacy of SAPT or DAPT after TAVR.

## 2 Method

This meta-analysis had applied for registration in the OSF REGISTRIES (10.17605/OSF.IO/Q42TE) international prospective register. The study will be performed in accordance with the Preferred Reporting Items for Systematic reviews and Meta-Analysis Protocols (PRISMA-P) guidelines.^[15,16]^

### 2.1 Search strategy

We will conduct a comprehensive systematic search to identify all relevant published studies from inception to January 10, 2021.^[17]^ There will be no restriction on the publication dates and languages. The following electronic databases will be searched: PubMed, EMBASE, The Cochrane Library, Cochrane Central Register of Controlled Trials. Search terms will combine MeSH and full text terms related to TAVR (transcatheter, percutaneous, aortic valve replacement, aortic valve implantation, TAVI, TAVR) and treatment (antithrombotic, antiplatelet, platelet aggregation inhibitors, aspirin, acetylsalicylic acid, acid acetylsalicylic, 2-(acetyloxy)benzoic acid, clopidogrel, prasugrel, ticagrelor, P2Y antagonist, P2Y receptor antagonist). We will perform manual search to identify additional publications from the reference lists of related reviews and meta-analyses.

### 2.2 Inclusion criteria

In this meta-analysis, we will include the articles that met the following criteria: patients with TAVR, comparison of DAPT with SAPT after TAVR, the primary outcome in the current study is all-cause mortality, while the secondary outcomes are bleeding (lethal, major, minor), stroke (major and minor), transient ischemic attack (TIA), spontaneous myocardial infarction (MI), valve thrombosis, vascular complications, and all clinical events are defined according to the Valve Academic Research Consortium-2,^[18]^ randomized controlled trials (RCTs) or observational studies. We will exclude the following articles: the studies are briefs, comments, letters, editorials, protocols, nonhuman studies; articles published in a language other than English and Chinese; published duplicate study.

### 2.3 Study selection

Two investigators will independently select the studies and any disagreement will be resolved by a third investigator through discussion. Titles and abstracts of the studies retrieved by the literature search will be screened base on inclusion/exclusion criteria and we will acquire the full text of potentially relevant studies for further assessment.

### 2.4 Data extraction

A standard form will be used to extract data from the included studies. Two investigators will independently extract the related data and any dispute will be discussed and resolved by a third investigator. To identify other relevant study data, we will contact the authors of published studies for incomplete data.

Extracted information will include: first author, publication year, journal, country of origin and funding source, sample size, mean age of participants, gender, specific condition, characteristics of interventions and comparators, overall mortality, bleeding, stroke, TIA, MI, valve thrombosis, vascular complications, the item of Risk of Bias (ROB), and the Newcastle-Ottawa Quality Assessment Scale (NOS).

### 2.5 Quality evaluation

Two reviewers will use the Cochrane ROB tool^[19,20]^ and NOS to assess the included studies independently. We will apply the Grades of Recommendation, Assessment, Development and Evaluation (GRADE) system to assess certainty of the evidence.^[21,22]^ The software, GRADEprofiler 3.6, is involved in implementing the scoring system of the evidence.

### 2.6 Statistical analysis and assessment of heterogeneity

Review Manager 5.3 software and Stata 15.0 (Stata Corporation, College Station, TX) will be used for general meta-analysis. We will use a random-effects model to pool the results of all studies and use the relative risk and 95% confidence intervals to summarize individual trial outcomes and estimate pooled effect. We will report the results narratively if data was not reported in a form that would allow inclusion in the meta-analysis. We will use Cochran test and the I^2^ statistic to evaluate the combination of heterogeneous studies. Where the number of studies for an outcome is sufficient (n≥10), a funnel plot will be used to examine for potential publication bias.

We will conduct subgroup analyses to investigate potential source of heterogeneity on treatment effect size, including clinical heterogeneity or methodological heterogeneity from different types of studies. Also, we plan to undertake subgroup analysis to examine effects of different comparisons, and populations when appropriate. When applicable, we will perform sensitivity analyses of results to look at the possible contribution of ROB such as randomization process, and concealment of random allocation.

## 3 Discussion

Undeniably, the concern for antithrombotic therapy after TAVR is of increasing importance, with a frequency of 1% to 5% for asymptomatic cases and much higher (40%) for symptomatic patients.^[23]^ Current clinical practice on antithrombotic therapy after TAVR was based on empirical and/or authority. Furthermore, there is no evidence on duration of therapy or what agents should be used. Despite the lack of evidence, the American guidelines and Canadian statement suggest aspirin indefinitely and clopidogrel for a specific time period.^[24,25]^

The existing systematic reviews have some limitations. First, the efficacy and safety cannot be evaluated with sufficient power due to small sample size. Second, because the original studies did not report the detailed relevant information, it is unable to perform subgroup analysis to investigate the various adverse events and evaluate the short mid and long-term outcomes of the 2 antiplatelet regimens. Finally, because of the significant variety in the baseline characteristics of the available study populations, direct comparison of the different antithrombotic regimens is challenging.

Therefore, we conducted a systematic review to explore the safety and efficacy of SAPT or DAPT after TAVR. Furthermore, the larger sample size RCTs are needed to provide further evidence.

## Author contributions

**Conceptualization:** Hongyan Li, Yafeng Wang.

**Data curation:** Hongyan Li, Yafeng Wang, Longyu Li, Bao Dan.

**Formal analysis:** Longyu Li, Bao Dan.

**Investigation:** Hongyan Li, Longyu Li.

**Methodology:** Hongyan Li, Yafeng Wang, Longyu Li, Bao Dan.

**Project administration:** Hongyan Li.

**Software:** Hongyan Li, Yafeng Wang.

**Supervision:** Hongyan Li.

**Writing – original draft:** Hongyan Li.

**Writing – review and editing:** Yafeng Wang, Longyu Li, Bao Dan.

## Abbreviations

Abbreviations: DAPT = dual-antiplatelet therapy, GRADE = Grades of Recommendation, Assessment, Development and Evaluation, MI = myocardial infarction, NOS = Newcastle-Ottawa Quality Assessment Scale, RCTs = randomized controlled trials, ROB = risk of bias, RR = relative risk, SAPT = single-antiplatelet therapy, TAVR = transcatheter aortic valve replacement, TIA = transient ischemic attack.
